# Loosening ER–Mitochondria Coupling by the Expression of the Presenilin 2 Loop Domain

**DOI:** 10.3390/cells10081968

**Published:** 2021-08-03

**Authors:** Michela Rossini, Paloma García-Casas, Riccardo Filadi, Paola Pizzo

**Affiliations:** 1 Department of Biomedical Sciences, University of Padua, 35131 Padua, Italy; michela.rossini@phd.unipd.it (M.R.); paloma.garciacasas@unipd.it (P.G.-C.); 2 Neuroscience Institute, National Research Council (CNR), 35131 Padua, Italy

**Keywords:** Presenilin 2, MAM, mitochondria, ER, Ca^2+^ signaling, organelle contacts, Alzheimer’s disease

## Abstract

Presenilin 2 (PS2), one of the three proteins in which mutations are linked to familial Alzheimer’s disease (FAD), exerts different functions within the cell independently of being part of the γ-secretase complex, thus unrelated to toxic amyloid peptide formation. In particular, its enrichment in endoplasmic reticulum (ER) membrane domains close to mitochondria (i.e., mitochondria-associated membranes, MAM) enables PS2 to modulate multiple processes taking place on these signaling hubs, such as Ca^2+^ handling and lipid synthesis. Importantly, upregulated MAM function appears to be critical in AD pathogenesis. We previously showed that FAD-PS2 mutants reinforce ER–mitochondria tethering, by interfering with the activity of mitofusin 2, favoring their Ca^2+^ crosstalk. Here, we deepened the molecular mechanism underlying PS2 activity on ER–mitochondria tethering, identifying its protein loop as an essential domain to mediate the reinforced ER–mitochondria connection in FAD-PS2 models. Moreover, we introduced a novel tool, the PS2 loop domain targeted to the outer mitochondrial membrane, Mit-PS2-LOOP, that is able to counteract the activity of FAD-PS2 on organelle tethering, which possibly helps in recovering the FAD-PS2-associated cellular alterations linked to an increased organelle coupling.

## 1. Introduction

Presenilin 1 and 2 (PS1, PS2) are two highly conserved, widely expressed, multi-pass transmembrane proteins mainly known as constituents of the catalytic core of the γ-secretase complex that is involved in the pathogenesis of Alzheimer’s disease (AD). The high molecular weight aspartyl-protease complex is formed by either PS1 or PS2 (cleaved into the N- and C-terminal fragments, NTF and CTF, respectively) and three other additional proteins, anterior pharynx defective 1 (APH1), presenilin enhancer (PEN-2) and nicastrin [1,2]. The enzymatic complex catalyses endomembranous proteolysis of different type I membrane proteins, with Amyloid Precursor Protein (APP) being the most studied γ-secretase substrate because of its involvement in the formation of amyloid β (Aβ) peptides. Indeed, the toxic effects of intracellular Aβ accumulation, as well as its extracellular deposition into Aβ plaques in the brain, are typical hallmarks associated with AD [3]. Importantly, different mutations in the genes encoding PS1, PS2 and APP are responsible for early-onset familial AD (FAD; [3]), indicating the crucial part played by the γ-secretase-dependent APP-processing pathway in disease onset. Accumulating evidence, however, supports the existence of additional and yet unidentified pathogenic mechanisms, further sustained by the fact that the majority of clinical trials targeting the Aβ pathway have failed to modify disease progression [4] (but see recent promising results for Lecanemab and Aducanumab; https://www.alzforum.org/therapeutics/lecanemab; accessed on 20 July 2021).

Beyond their well-defined catalytic role within the γ-secretase complex, both PS1 and PS2, as individual proteins, exert specific activities that modulate additional important cell pathways, such as protein trafficking, cell adhesion, mitochondrial metabolism and Ca^2+^ homeostasis [5]. The latter is differently modulated by FAD-PS1 and FAD-PS2 and is also altered in sporadic forms of the disease [6,7,8,9,10]. As far as PS2 is concerned, we previously described its action on multiple Ca^2+^ signalling pathways, independently of γ-secretase activity and with disease-associated forms being more effective than the wild-type (WT) protein [11,12]. In particular, FAD-linked PS2 mutants have been shown to reduce the Ca^2+^ content of intracellular stores, mainly the endoplasmic reticulum (ER) and the Golgi apparatus [13,14,15], resulting in blunted cytosolic Ca^2+^ rises induced by IP3-generating cell stimulations (or by inhibiting the SERCA pump) [13,14,16,17,18,19,20]. These effects depend on the capacity of PS2 to interfere with SERCA activity, partially blocking it [21], and induce hyperactivation of IP3 receptors (IP3Rs) by increasing their sensitivity to IP3 [22,23]. Moreover, FAD-PS2 mutants decrease Store-Operated Ca^2+^ Entry (SOCE; [24]) by reducing STIM1 levels [15], further contributing to the lower ER Ca^2+^ content found in FAD-PS2-expressing cells. Importantly, PS2 mutants also alter key mitochondrial functions. As a consequence of a reduced ER Ca^2+^ content and blunted IP3R-mediated Ca^2+^ release upon cell stimulation, mitochondrial Ca^2+^ uptake is also decreased in FAD-PS2-expressing cells, impacting on organelle bioenergetics [20]. Finally, PS2, both WT and FAD mutants, modulate the physical and functional interaction of mitochondria with the ER [18,19] by binding to mitofusin 2 (MFN2), a master regulator of ER–mitochondria tethering [25]. The interface domain between the two organelles, also defined as MAM (mitochondria-associated membranes; [26]), represents an intracellular signalling hub where different processes take place, such as lipid synthesis, apoptosis, autophagy and Ca^2+^ handling [27,28]. Importantly, several disease-associated proteins, including PS [19,29], have been found enriched in MAM, being able to alter functional organelle crosstalk [27,30], and strongly indicating the importance of this domain for cell pathophysiology.

Considering the potential key role of altered ER–mitochondria interactions in neuronal dysfunction and AD onset, we here investigated which PS2 protein domain exerts the modulatory effect on organelle coupling. We found that PS2-CTF mimics the effects of the full-length protein, whereas the expression, free in the cytosol, of just the cytosolic loop located between the sixth and the seventh transmembrane domains (TMD) of the protein (PS2-LOOP, also contained within PS2-CTF), does not. However, when the PS2-LOOP domain was expressed at the mitochondrial surface to also force its presence in MAM (where it is physiologically enriched, facing mitochondria, while expressed as part of the endogenous PS2 protein, though being anchored to the ER membrane), it binds to MFN2 and counteracts the PS2 action on ER–mitochondria coupling, thereby reducing organelle Ca^2+^ transfer and tethering. Importantly, when expressed in FAD-PS2 patient-derived fibroblasts, the mitochondrial PS2-LOOP normalizes ER–mitochondria tethering and rectifies the increased lipid droplet formation found in AD cells, strongly supporting its antagonist action towards FAD-PS2.

Altogether these data define the PS2-LOOP as the critical domain of the protein mediating its interaction with MFN2, thus being crucial for the activity of PS2 on ER–mitochondria coupling. Moreover, they suggest the possibility to use this mitochondria-targeted peptide as a tool to counteract possible deleterious effects triggered by FAD-PS2 through alterations of the ER–mitochondria interface.

## 2. Materials and Methods

### 2.1. Plasmids

Most of the cDNAs used in this work were already described before: PS2 WT [18], Cyt-GFP (pEGFP-C1, Clontech), Mit-GFP, cytosolic and mitochondrial aequorin [31], and SPLICSs [32].

The PS2-CTF was amplified from a plasmid bearing the cDNA of PS2 WT with the following primers (5′-3′):

Fw: CGCGGATCCATGGACTACAAAGACGATGACGACAAGAAAGGGCCTCTGAGA

Rv: CCGGAATTCTCACAGGTCTTCTTCAGAGATCAGTTTCTGTTCGATGTAGAGCTGATGGGA

The PCR product was digested with the BamHI and EcoRI restriction enzymes (FastDigest, Thermo Scientific) and ligated into pcDNA3. 

To design Cyt-PS2-LOOP, Cyt-CTRL, Mit-PS2-LOOP and Mit-CTRL, the cDNA of tdTomato was amplified from the tdTomato-C1 plasmid (a gift from Michael Davidson, Addgene plasmid # 54653), using the following primers (5′-3′):

Fw: CGCGGTACCATGGTGAGCAAGGGC 

Rv: GCGGATATCCTTGTACAGCTCGTCCATG

The PCR product was then digested using KpnI and EcoRV restriction enzymes (Thermo Scientific FastDigest). 

The PS2-LOOP was amplified from a plasmid bearing the cDNA of PS2 WT with the following primers (5′-3′):

Fw: CGCGATATCGGTGGAGGTGGAGGTGACTACAAAGACGACGACGACAAGATGGCGAAGCTGGACC

Rv: GGCCTCGAGTTAGCGGCCGCTCTTCACGCCCCTTTC

In the forward primer, before the region annealing with PS2-LOOP, a disordered linker was inserted (containing a FLAG-tag) to allow proper folding. 

The PCR product was digested using EcoRV and XhoI restriction enzymes (Thermo Scientific).

The mitochondrial targeting sequence of AKAP1 was amplified from a previously described AKAP1-bearing plasmid (OMM-RFP, a gift from G. Hajnoczky, [33]) using the following primers (5′-3′):

Fw: CGCAAGCTTGCCACCATGG

Rv: GCGGGTACCGCGCCCACTGCCTTTTCCTTTTCCAGATCCACCAGATTTAGATAGGATAGCACCAGC

In the reverse primer, a disordered linker was included to permit all portions of the construct to fold independently. 

The PCR product was digested using HindIII and KpnI restriction enzymes (Thermo Scientific). The digested products were ligated in a pcDNA3 vector.

To generate ER-tdTomato, the ER targeting sequence from CYP450-2C1, which anchors the fusion protein to the ER membranes [34], was amplified from the ER-ABKAR construct (a gift from Takanari Inoue and Jin Zhang, Addgene plasmid # 61508) with the following primers (5′-3′): 

Fw: CGCAAGCTTGCCACCATGGACCCTGTGGTG

Rv: GCCGATATCGGTACCTCCAGATCCACCAGACCCTCCCCCATAGC

In the reverse primer, a disordered linker was included to permit all portions of the construct to fold independently. 

### 2.2. Cell Culture and Transfection

HeLa and SH-SY5Y cells were maintained in DMEM (Sigma-Aldrich, D5671) supplemented with 10% FCS, 2 mM L-glutamine, 100 U/mL penicillin and 0.1 mg/mL streptomycin. Cells were grown in a humidified Heraeus incubator at 37 °C, with 5% dissolved CO_2_. 

Cells were seeded 24–48 h before transfection to reach 50–60% confluence at the moment of transfection, performed with either TransIT-LT1 (Mirus Bio, HeLa cells) or LipofectamineTM 2000 (Thermo Fisher, SH-SY5Y cells) following the manufacturer’s instructions. 

Primary human skin fibroblasts from a healthy donor (82-year-old, female) and a FAD-PS2-N141I patient (81-year-old, female) were obtained from the Coriell Institute for medical research (respectively, AG08269 and AG09908) and grown in DMEM (Sigma-Aldrich, D5671) supplemented with 15% FCS, 2 mM L-glutamine, 100 U/mL penicillin and 0.1 mg/mL streptomycin in a humidified Heraeus incubator at 37 °C with 5% dissolved CO_2_). For transfection, fibroblasts were electroporated with the Neon transfection kit (Thermo scientific) following the manufacturer’s instructions. A single pulse (1600 V, 20 ms) was performed before seeding cells.

### 2.3. Protein Extraction and Western Blot Analysis

Twenty-four hours after transfection, SH-SY5Y cells expressing either Cyt-PS2-LOOP, Cyt-CTRL, Mit-PS2-LOOP or Mit-CTRL were washed with PBS and lysed in RIPA buffer (50 mM Tris, 150 mM NaCl, 1% Triton X-100, 0.5% deoxycholic acid, 0.1% SDS, protease and phosphatase inhibitor cocktails; Roche, 11836170001 and 4906845001, respectively, pH 7.5). The lysates were incubated on ice for 30 min and the supernatants were collected after being spun down at 13,000 rcf for 15 min at 4 °C. Then, 40 μg of the proteins were separated by SDS-PAGE, blotted and probed with the following antibodies: α-tdTomato (OAEA00012, Aviva System Biology), α-actin (A4700, Sigma-Aldrich), α-MFN2 (ab56889, Abcam), and α-PS2 (1987-1, Epitomics). ECL (Amersham, GE Healthcare) was used to detect chemiluminescence using an Uvitec Mini HD9 apparatus (Eppendorf). The intensity of the bands was then measured by ImageJ (NIH).

### 2.4. Immunoprecipitation

Proteins were extracted from SH-SY5Y cells expressing Mit-CTRL or Mit-PS2-LOOP in modified RIPA buffer (150 mM NaCl, 25 mM Tris-HCl, 1% NP40, 0.01% SDS, 0.05% Na-DOC, 1 mM Na_2_EDTA, protease inhibitor cocktail, pH 7.4). For each lysate, 500 μg was incubated overnight at 4 °C on a rocker platform with 1 µg of α-MFN2 antibody (ab56889 Abcam) or with 1 µg of an unrelated α-HA antibody (Roche), as a negative control (IgG). Samples were then adsorbed with 25 μL of protein A/G-Agarose beads (sc-2003, Santa Cruz Biotechnology) at 4 °C for 3 h, centrifuged at 2000 rcf and washed 4x in 0.1 M NaCl supplemented with a protease inhibitor cocktail (11836170001 Roche). Beads were resuspended in 40 μL of Loading Buffer and incubated at 95 °C for 10 min. Proteins were separated by SDS-PAGE, blotted on a nitrocellulose membrane and probed with either α-tdTomato (OAEA00012, Aviva System Biology) or α-MFN2 (ab56889, Abcam) antibodies.

### 2.5. Aequorin Ca^2+^ Measurements

HeLa and SH-SY5Y cells (0.5 × 10^5^) were plated on 13 mm coverslips and transfected after 24 h with either cytosolic- or mitochondrial-aequorin encoding plasmids (0.5 µg) together with the indicated plasmids (1 µg), as indicated above. Human fibroblasts were plated after electroporation (see above). Twenty-four hours after transfection/electroporation, cells were incubated at 37 °C with 5 µM native coelenterazine (Biotium) in mKRB (135 mM NaCl, 5 mM KCl, 1 mM MgCl_2_, 1 mM CaCl_2_, 20 mM HEPES, 11 mM glucose, pH 7.4 at 37 °C). Cells were transferred to the perfusion chamber and the experiments were performed in mKRB at 37 °C, collecting photons with a Photon counter (9125, Sens-Tech). Bradykinin (100 nM), histamine (100 μM) or FCS (3%) were added in Ca^2+^-free EGTA (500 μM)-containing mKRB, together with the SERCA inhibitor cyclopizonic acid (CPA, 10 µM, to avoid Ca^2+^ reuptake in the ER). At the end of the experiments, cells were lysed with digitonin (100 µM) in a hypotonic, Ca^2+^-rich solution (10 mM CaCl_2_ in H_2_O) to consume the remaining unused aequorin pool and calibrate the experiment. The signal was converted into [Ca^2+^] as previously described [35].

Where indicated, to partially pre-deplete control cells of their ER Ca^2+^ content, cells were treated for 120 s in Ca^2+^-free EGTA-containing mKRB supplemented with CPA (10 µM), before stimulation with BK. 

To induce SOCE, after stimulation with BK + CPA (as described above), cells were bathed for 6 min in Ca^2+^-free, EGTA (50 µM)-containing mKRB supplemented with CPA (10 µM), to completely deplete ER Ca^2+^ content. Ca^2+^ entrance was induced by cell perfusion with mKRB supplemented with CaCl_2_ (2 mM) and CPA (10 µM).

### 2.6. Immunofluorescence and Confocal Microscopy 

Twenty-four hours after transfection, cells were washed with PBS and fixed with PFA 4% (in PBS) for 10 min. Cells were washed 3 × 5 min in PBS in gentle agitation, incubated 20 min in quenching solution (0.24% NH_4_Cl in PBS), washed 3 × 5 min in PBS and either mounted with Mowiol (SIGMA) or further processed for immunofluorescence (IF) or lipid droplet staining, as detailed below.

For IF, after fixation, cells were treated with a permeabilization solution (0.1% Triton in PBS) for 3 min, washed with PBS and incubated 30 min in blocking solution (2% BSA, 0.1% gelatin, 10% goat serum in PBS). Coverslips were incubated for 1 h (RT) with α-MYC tag antibody (05-724, Millipore) or α-TOM20 antibody (sc-17764 for HeLa cells and sc-11415 for SH-SY5Y cells, Santa Cruz Biotechnology) diluted in blocking solution (1:150, 1:100 and 1:100, respectively), washed 3 × 5 min with blocking solution in gentle agitation and incubated with the secondary antibody (1:400 in blocking solution, Alexa-488 donkey anti-mouse or anti-rabbit, Thermo Fisher) for 45 min (RT). After 3 × 5 min washes in blocking solution, coverslips were mounted with Mowiol (SIGMA). For lipid droplet staining, after fixation, cells were treated with HCS LipidTOX™ Green Neutral Lipid Stain (H34475, Thermo Fisher) for 30 min according to the manufacturer’s instructions.

Images were collected with a Leica SP5 confocal microscope (DM IRE2) by WLL laser, acquiring the different colour channels independently. Alexa 488, cytosolic and mitochondrial GFP, along with SPLICSs, were excited at 488 nm, whereas for TdTomato it was at 555 nm.

The collected images were analysed by ImageJ or Fiji (NIH). To evaluate lipid droplet or SPLICSs staining, multi-stack images were collected, keeping the same parameters during acquisition (zoom, laser intensity, PMT gain). For SPLICSs analysis, images were background-subtracted and the Convolve filter was applied to minimize dot fragmentation. Dots were counted using the 3D Objects Counter plugin, considering only objects in the range 5–500 pixels, to avoid recording noise or aggregates, respectively.

Stained lipid droplets were excited at 485 nm and their number, volume and surface were calculated through all stacks by the 3D Objects Counter plugin after calculating a threshold (corresponding to 2× the mean fluorescence intensity of each cell), considering only objects in the range 5–500 pixels.

The mitochondrial morphological analysis was performed on single-stack images subtracting the background. Mean and Noise Despeckle ImageJ plugins were applied and a binary image was obtained by setting an automatic threshold, to better resolve the mitochondrial network. Mitochondrial mean area, perimeter, circularity and aspect ratio values were calculated by the Analyze Particle ImageJ plugin, applying a cut-off of 3 pixels. 

In the representative images, after quantification performed as detailed above, the signals were enhanced with the automatic ImageJ plugins Brightness/Contrast and Enhance Image, to better appreciate low fluorescent signals.

### 2.7. Statistical Analysis

All data are representative of at least 3 independent experiments. Significance was calculated by unpaired Student’s t-test for normal distributions and Wilcoxon Mann–Whitney test for not normally distributed data. * = *p* < 0.05, ** = *p* < 0.01, *** = *p* < 0.001. Values are reported as mean ± SEM. For additional information, see Table S1.

## 3. Results

### 3.1. The PS2 C-Terminal Fragment, but Not Its Loop Domain, Retains the Effects of Full-Length PS2 on ER–Mitochondria Ca^2+^ Transfer

**Figure 1:**
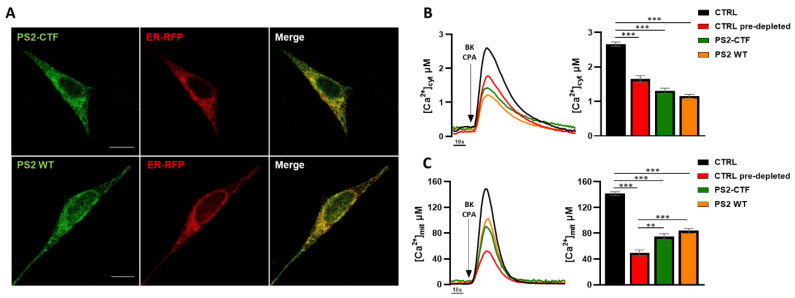
PS2-CTF retains the effects of full-length PS2 on ER–mitochondria Ca^2+^ transfer. (**A**) Confocal images of SH-SY5Y cells co-expressing either PS2-CTF or PS2 WT and an RFP targeted to the ER (ER-RFP). Scale bar, 10 µm. (**B**,**C**) Representative cytosolic (**B**) and mitochondrial (**C**) Ca^2+^ traces in control (black), pre-depleted control (red), PS2-CTF- (green) and PS2 WT- (orange) expressing SH-SY5Y cells stimulated with BK (100 nM) and CPA (10 µM). On the right of each panel, the bar graph represents the mean [Ca^2+^] peak values obtained upon cell stimulation (mean ± SEM; number of independent experiments: cytosolic data: CTRL n = 5; CTRL pre-emptied n = 15; PS2-CTF n = 8; PS2-WT n = 7; mitochondrial data: CTRL n = 5; CTRL pre-depleted n = 11; PS2-CTF n = 9; PS2-WT n = 9). **= *p* < 0.01, *** = *p* < 0.001.

To define the PS2 domain mediating the effects of the protein on ER–mitochondria Ca^2+^ shuttling, we expressed in SH-SY5Y cells the PS2-CTF (aa 298-448, physiologically generated upon the autoproteolytic cleavage of the full-length protein [36]) fused to a Myc-tag, as well as the entire Myc-tagged-PS2, for comparison. Immunofluorescence staining revealed that both PS2 and PS2-CTF localize in endomembranes, mostly in the ER (Figure 1A). Importantly, upon maximal IP3R-dependent ER Ca^2+^ release, the expression of either PS2 or PS2-CTF induced a similar decrease in cytosolic Ca^2+^ peaks, compared to control cells (Figure 1B). We previously reported that this phenomenon depends on a reduced ER Ca^2+^ content induced by PS2 expression [14,15,21], suggesting that PS2-CTF conserves the same effect. On this line, since mitochondrial Ca^2+^ uptake is deeply affected by the amount of Ca^2+^ released from the ER through IP3Rs, lower mitochondrial Ca^2+^ peaks were observed in PS2- or PS2-CTF-expressing cells (Figure 1C), compared to controls.

To better investigate the process of mitochondrial Ca^2+^ uptake independently of the different Ca^2+^ levels reached in the cytosol upon cell stimulation, the amplitude of cytosolic Ca^2+^ peaks in control cells were matched with those observed in PS2- or PS2-CTF-expressing cells by a pre-depletion protocol (CTRL pre-depleted, Figure 1B; see Materials and Methods). Notably, the mitochondrial Ca^2+^ peaks in PS2- or PS2-CTF-expressing cells were substantially increased compared to those of pre-depleted controls (Figure 1C), hinting at a higher efficiency of ER–mitochondria Ca^2+^ shuttling. This is in line with our previous finding of increased physical and functional ER–mitochondria tethering in cells expressing PS2, in particular FAD-PS2 mutants [18,19], again indicating that PS2-CTF exerts a similar function to the full-length protein.

**Figure 2:**
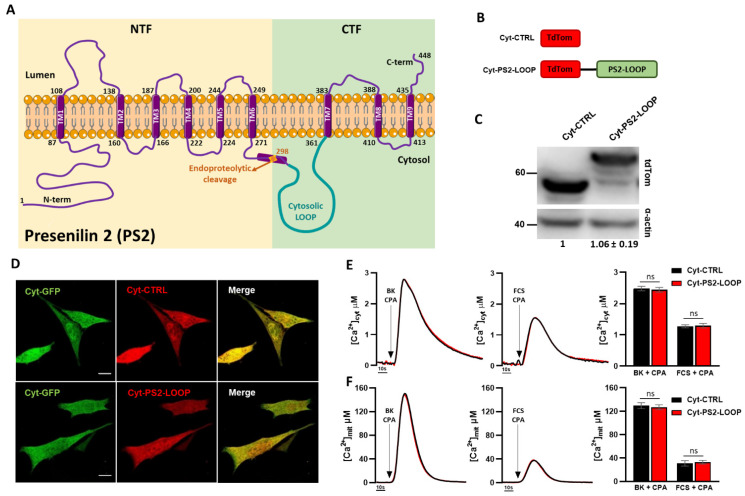
Cyt-PS2-LOOP does not affect ER–mitochondria Ca^2+^ handling. (**A**) PS2 topology. PS2 is a 9 TMD protein with an endoproteolytic cleavage site at the aa 298, that leads to the generation of an N-terminal (NTF) and a C-terminal (CTF) fragment. The cytosolic loop (PS2-LOOP, aa 305-361; highlighted in green) is retained within the CTF. (**B**) The cartoon represents the specific protein domains within the Cyt-CTRL and Cyt-PS2-LOOP constructs. (**C**) Representative WB of SH-SY5Y cells, expressing either Cyt-CTRL or Cyt-PS2-LOOP, decorated with α-tdTomato antibody, showing a similar expression for both constructs (1 vs. 1.06 ± 0.19, mean ± SEM, n = 3 independent experiments per condition). α-actin is also shown as the loading control. (**D**) Confocal images of SH-SY5Y cells co-expressing either Cyt-CTRL or Cyt-PS2-LOOP and a cytosolic GFP (Cyt-GFP). Scale bar, 10 µm. (**E, F**) Representative traces of cytosolic (**E**) and mitochondrial (**F**) Ca^2+^ in SH-SY5Y cells expressing either Cyt-CTRL (black) or Cyt-PS2-LOOP (red) upon stimulation with BK (100 nM) and CPA (10 µM), or FCS (3%) and CPA (10 µM). On the right of each panel, the graph bar represents the mean cytosolic (**E**) or mitochondrial (**F**) [Ca^2+^] peak values obtained in the different conditions (mean ± SEM; number of independent experiments: cytosolic data: Cyt-CTRL (BK + CPA) n = 12; Cyt PS2-LOOP (BK + CPA) n = 12; Cyt-CTRL (FCS + CPA) n = 9; Cyt-PS2-LOOP n = 9; mitochondrial data: Cyt-CTRL (BK + CPA) n=10; Cyt-PS2-LOOP (BK + CPA) n = 11; Cyt-CTRL (FCS + CPA) n = 7; Cyt-PS2-LOOP n = 8). No significant differences were appreciated in any condition (ns).

We previously demonstrated that the activity of PS2 on ER–mitochondria coupling depends on its binding to MFN2 [19]. Moreover, we reported that PS2-CTF, as well as a truncated form of PS2 (PS2-Δ374–448, lacking the final part of PS2-CTF but containing its large cytosolic loop (PS2-LOOP; Figure 2A), interacts with MFN2, whereas the PS2-NTF does not [19]. These data suggest that PS2-LOOP might be critical for PS2-MFN2 interaction and the potentiation of ER–mitochondria coupling. We therefore tested whether the expression of PS2-LOOP alone (PS2 aa 305-361; Figure 2A), without the other PS2 protein domains, affects organelle coupling. To visualize its subcellular localization, PS2-LOOP was fused with the fluorescent protein tdTomato (Cyt-PS2-LOOP, Figure 2B); a construct encoding only the tdTomato (Cyt-CTRL) was also generated, as a control (Figure 2B). Importantly, both constructs are efficiently expressed at comparable levels, as revealed by Western Blot (WB) analysis (Figure 2C) and present a similar intracellular distribution, as shown by the co-expression of either Cyt-CTRL or Cyt-PS2-LOOP with a cytosolic GFP in SH-SY5Y cells (Figure 2D).

We then evaluated whether Cyt-PS2-LOOP maintains an effect on ER–mitochondria Ca^2+^ transfer. When expressed in SH-SY5Y cells, no significant differences in the cytosolic (Figure 2E) or in the mitochondrial (Figure 2F) Ca^2+^ peaks (obtained by either bradykinin (BK) or fetal calf serum (FCS), to induce respectively, a maximal or milder, more physiological, IP3R-dependent ER Ca^2+^ release [20]), were observed compared to control cells. The lack of significant effects on cellular Ca^2+^ handling suggests that Cyt-PS2-LOOP, when expressed free in the cytosol, does not maintain the capacity of PS2 (as well as of PS2-CTF; see Figure 1C) to impact on ER–mitochondria coupling. 

### 3.2. The Mitochondria-Targeted PS2 Loop Domain Exerts an Opposite Effect Compared to Full-Length PS2 on ER–Mitochondria Coupling

**Figure 3:**
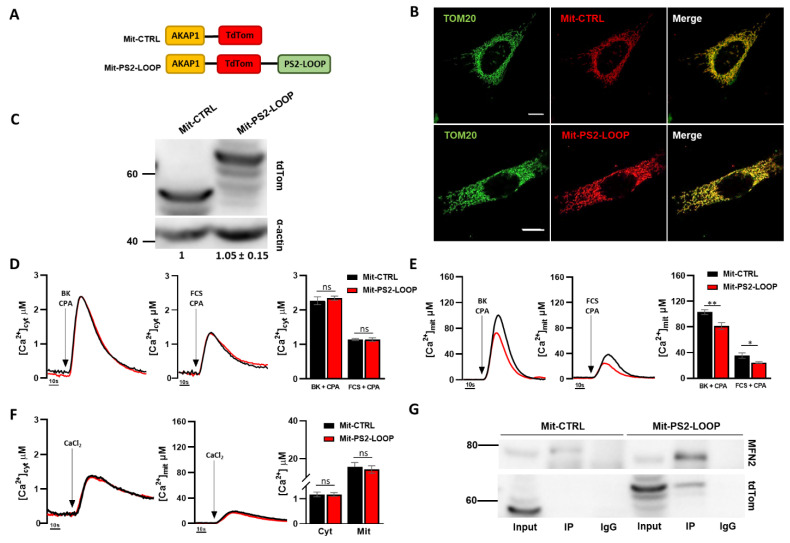
Mit-PS2-LOOP exerts an opposite effect on ER–mitochondria coupling compared to full-length PS2. (**A**) The cartoon represents the specific protein domains within the Mit-CTRL and Mit-PS2-LOOP constructs. (**B**) Confocal images of SH-SY5Y cells expressing either Mit-CTRL or Mit-PS2-LOOP, immunostained with α-TOM20 antibody. Scale bar, 10 µm. (**C**) Representative WB of SH-SY5Y cells, expressing either Mit-CTRL or Mit-PS2-LOOP, decorated with α-tdTomato antibody, showing a similar expression of both constructs (1 vs. 1.05 ± 0.15). α-actin is also shown as loading control. (**D, E**) Representative traces of cytosolic (**D**) or mitochondrial (**E**) Ca^2+^ rises in SH-SY5Y cells expressing either Mit-CTRL (black) or Mit-PS2-LOOP (red) upon stimulation with BK (100 nM) and CPA (10 µM) or FCS (3%) and CPA (10 µM). On the right of each panel, the graph bar represents the mean cytosolic (**D**) or mitochondrial (**E**) [Ca^2+^] peak values in the different conditions (mean ± SEM; number of independent experiments: cytosolic data: Mit-CTRL (BK + CPA) n=11; Mit PS2-LOOP (BK + CPA) n = 12; Mit-CTRL (FCS + CPA) n = 13; Cyt-PS2-LOOP n=12; mitochondrial data: Mit-CTRL (BK + CPA) n = 11; Mit-PS2-LOOP (BK + CPA) n = 10; Mit-CTRL (FCS + CPA) n = 11; Mit-PS2-LOOP n = 11). ns, not significant. (**F**) Representative cytosolic (left) and mitochondrial (right) traces of Ca^2+^ rises in SH-SY5Y cells expressing either Mit-CTRL (black) or Mit-PS2-LOOP (red) induced by SOCE activation, i.e., Ca^2+^ (2 mM) re-addition following a complete store depletion induced by BK (100 nM) and CPA (10 µM) exposure (see Methods). The graph bar represents the mean cytosolic and mitochondrial [Ca^2+^] peak values in the different conditions (mean ± SEM; number of independent experiments: cytosolic data: Mit-CTRL n = 10; Mit-PS2-LOOP n = 10; mitochondrial data: Mit-CTRL n = 11; Mit-PS2-LOOP n = 12). ns, not significant. (**G**) Representative WB of co-IP experiments performed in SH-SY5Y cells expressing either Mit-CTRL or Mit-PS2-LOOP. MFN2 was precipitated by a specific monoclonal antibody, and the coprecipitated samples were analyzed by SDS-PAGE and probed with α-tdTomato and α-MFN2 antibodies, as indicated. In the negative controls, an irrelevant antibody (IgG) was used for the IP (n = 3 independent experiments).

Since PS2 (as well as PS2-CTF) is enriched in MAM [19,29], whereby its binding to MFN2 and its activity on organelle tethering might be favoured by an advantageous stoichiometry, we reasoned that the PS2 loop domain could also benefit from a proper localization/enrichment. To test this hypothesis, we therefore targeted, by an appropriate targeting sequence (see Materials and Methods), the fused protein tdTomato-PS2-LOOP (Mit-PS2-LOOP), or the tdTomato protein alone (Mit-CTRL) as a control, to the outer mitochondrial membrane (OMM) (Figure 3A). Both Mit-CTRL and Mit-PS2-LOOP correctly localized to the mitochondrial network (Figure 3B and Figure S1A) and were expressed at similar levels within cells (Figure 3C). Upon BK- or FCS-induced stimulation of SH-SY5Y cells, the cytosolic Ca^2+^ peaks that resulted were unaffected by the expression of MIT-PS2-LOOP (Figure 3D). Importantly, however, the corresponding mitochondrial Ca^2+^ peaks, obtained by the same stimulations, were significantly lower in cells expressing Mit-PS2-LOOP, compared to those observed in Mit-CTRL-expressing cells (Figure 3E), suggesting a reduced efficiency of ER–mitochondria Ca^2+^ transfer in this condition, and implying an opposite effect of Mit-PS2-LOOP with respect to the full-length PS2 protein or its CTF (see Figure 1C). Indeed, the reduced mitochondrial Ca^2+^ rises induced by Mit-PS2-LOOP expression do not depend on an intrinsically defective capacity of mitochondria to take up Ca^2+^, as revealed by the similar cytosolic and mitochondrial Ca^2+^ peaks observed upon activation of store-operated Ca^2+^ entry (SOCE; Figure 3F), i.e., a condition in which cytosolic and mitochondrial Ca^2+^ elevations are induced by cation entry through the plasma membrane, rather than by its release from the ER.

A similar reduction in IP3R-dependent mitochondrial Ca^2+^ peaks was observed in Mit-PS2-LOOP-expressing HeLa cells (Figure S1B). Importantly, by a recently reported sensor for close (<8–10 nm) ER–mitochondria contacts (SPLICSs; [32]), a lower number of dots (representing organelle close contacts) was retrieved in Mit-PS2-LOOP-expressing cells, compared to controls (Figure S1C), suggesting that a reduced ER–mitochondria tethering decreases the efficiency of Ca^2+^ shuttling between the two organelles. Of note, mitochondrial morphology was not affected by Mit-PS2-LOOP expression (Figure S1D). 

We previously demonstrated that PS2 modulates ER–mitochondria tethering by binding to MFN2 and tuning its negative activity on inter-organelle coupling [19]. We therefore speculated that Mit-PS2-LOOP might compete with endogenous, WT or mutated PS2 for MFN2 binding, thus triggering opposite effects on ER–mitochondria coupling. In line with this hypothesis, we found that Mit-PS2-LOOP, but not Mit-CTRL, coimmunoprecipitates with endogenous MFN2 (Figure 3G).

### 3.3. The Mitochondria-Targeted PS2 Loop Domain Normalizes ER–Mitochondria Tethering in FAD-PS2-N141I Patient-Derived Fibroblasts

We previously demonstrated that FAD-PS2-N141I patient-derived fibroblasts show increased ER–mitochondria tethering and functional Ca^2+^ transfer compared to control cells obtained from age-matched healthy individuals [19], indicating that mutated PS2 potentiates organelle coupling at endogenous levels of expression. Both WT and FAD-PS2 forms interact with MFN2, with an increased efficiency of FAD-PS2 because of its enrichment in MAM, compared to the WT counterpart [19]. 

**Figure 4:**
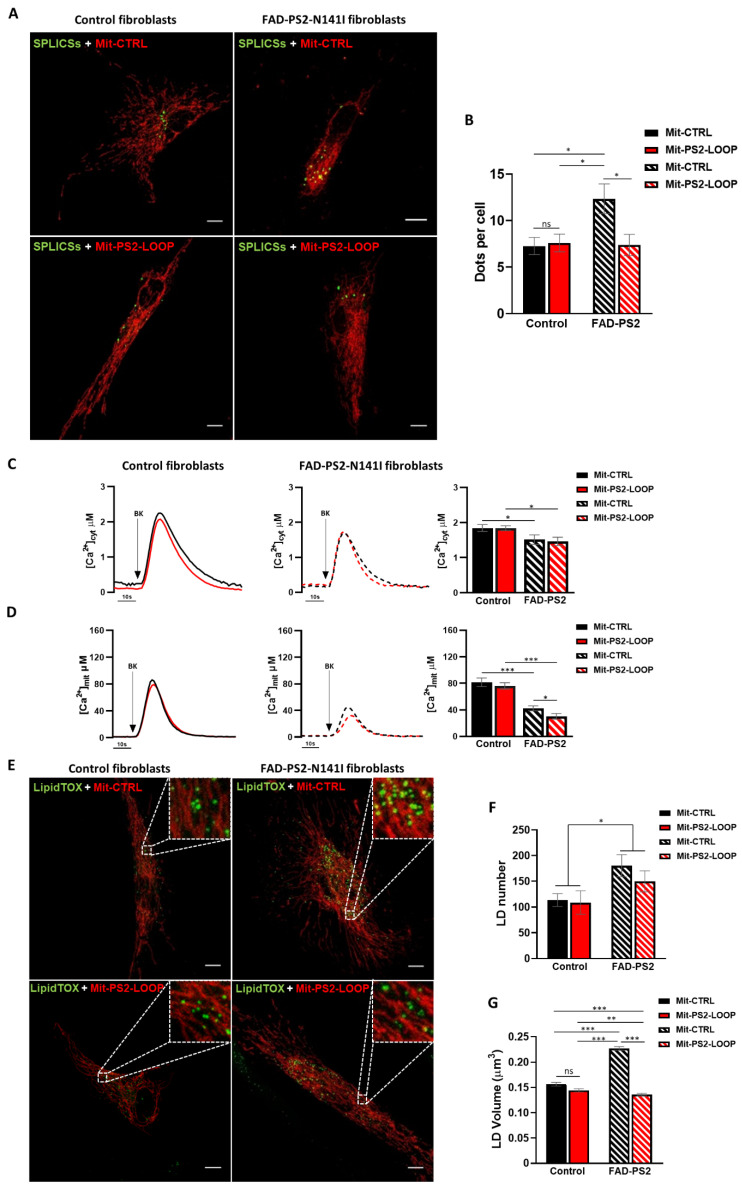
Mit-PS2-LOOP normalizes ER–mitochondria coupling in FAD-PS2-N141I patient-derived fibroblasts. (**A**) Confocal images of control and FAD-PS2-N141I patient-derived fibroblasts co-expressing either Mit-CTRL or Mit-PS2-LOOP (red) and the SPLICSs probe (green), to asses ER–mitochondria close contacts. Scale bar, 10 µm. (**B**) The graph bar represents the mean value of SPLICSs dots per cell in the different conditions (mean ± SEM; Mit-CTRL (Control) n = 15; Mit-PS2-LOOP (Control) n = 20; Mit-CTRL (FAD-PS2) n = 24; Mit-PS2-LOOP (FAD-PS2) n = 16, from 3 independent experiments. ns, not significant). (**C, D**) Representative traces of cytosolic (**C**) and mitochondrial (**D**) Ca^2+^ rises in control and FAD-PS2-N141I patient-derived fibroblasts expressing either Mit-CTRL (black) or Mit-PS2-LOOP (red), induced by BK (100 nM). On the right of each panel, the graph bar represents the mean cytosolic (**C**) and mitochondrial (**D**) [Ca^2+^] peak values in the different conditions (mean ± SEM; number of independent experiments: cytosolic data: Mit-CTRL (Control) n = 9; Mit-PS2-LOOP (Control) n = 13; Mit-CTRL (FAD-PS2) n = 7; Mit-PS2-LOOP (FAD-PS2) n = 6; mitochondrial data: Mit-CTRL (Control) n = 19; Mit-PS2-LOOP (Control) n = 20; Mit-CTRL (FAD-PS2) n = 13; Mit-PS2-LOOP (FAD-PS2) n = 16). (**E**) Confocal images of control and FAD-PS2-N141I patient-derived fibroblasts expressing either Mit-CTRL or Mit-PS2-LOOP (red) and stained with LipidTOX (green) to asses LDs. Scale bar, 10 µm. (**F, G**) The graph bars represent the mean value of either LD number per cell (**F**) or LD volume (µm^3^; **G**) in the different conditions (mean ± SEM; n = 36–40 cells from 3 independent experiments; n = 3900–7000 LDs. ns, not significant). * = *p* < 0.05, ** = *p* < 0.01, *** = *p* < 0.001.

We thus wondered whether Mit-PS2-LOOP could be able to hamper the PS2-N141I-mediated ER–mitochondria tethering in FAD patient-derived fibroblasts. To this purpose, both ER–mitochondria contact sites and organelle Ca^2+^ transfer were investigated in control and FAD-PS2-N141I fibroblasts, expressing either Mit-PS2-LOOP or Mit-CTRL. The SPLICSs probe was expressed in both control and FAD-PS2-N141I fibroblasts to detect ER–mitochondria close contacts (Figure 4A). As already reported [32], FAD-PS2-N141I patient-derived cells showed an increased number of SPLICSs-dependent dots (Figure 4B), confirming the increased ER–mitochondria tethering found in different FAD-PS2 cell models [14,18,19], compared to controls. Importantly, the expression of Mit-PS2-LOOP in FAD-PS2-N141I fibroblasts fully corrects the parameter, lowering the number of SPLICSs dots to control values (Figure 4B). 

As to organelle Ca^2+^ handling, the BK-induced maximal ER Ca^2+^ release caused blunted cytosolic Ca^2+^ rises in FAD-PS2-N141I fibroblasts compared to controls (Figure 4C), confirming the reduced ER Ca^2+^ content found in these and other FAD-PS2-expressing cells [13,14,15,19]. Consequently, mitochondrial Ca^2+^ rises induced by the same stimulation were also reduced in FAD-PS2 patient-derived cells compared to controls (Figure 4D). In accordance with ER–mitochondria tethering data (Figure 4A-B and Figure S1C), Mit-PS2-LOOP expression induced a further decrease in mitochondrial Ca^2+^ rises of FAD-PS2-N141I fibroblasts (Figure 4D), because its expression keeps the two organelles further apart, thus making their Ca^2+^ transfer less efficient. 

### 3.4. The Mitochondria-Targeted PS2 Loop Domain Rectifies the Increased Lipid Droplet Formation Found in FAD-PS2-N141I Patient-Derived Fibroblasts

Lipid droplets (LDs) are key ubiquitous eukaryotic organelles providing essential lipid precursors for membrane biogenesis (thus supporting organelle and cell growth) and acting as a sink for toxic fatty acids. They are connected to different organelles within the cells [37], being also identified in the proximity of both ER and mitochondria [38]. Interestingly, an increased LD formation has been linked to an altered ER–mitochondria tethering in sporadic and familial AD patient-derived fibroblasts [39]. We thus wondered whether the Mit-PS2-LOOP, being able to modulate ER–mitochondria tethering in FAD-PS2 cells, could also modify the formation of these structures in FAD patient-derived fibroblasts.

We first visualized LDs in control and FAD-PS2-N141I fibroblasts, finding an increase in the number and volume of these organelles in diseased cells, compared to controls (Figure 4E–G), and in line with previous results [39]. Of note, when the Mit-PS2-LOOP was expressed, a reduction, although not significant, in LD number and a complete recovery in LD size was observed in FAD-PS2 fibroblasts (Figure 4F,G).

## 4. Discussion

The dynamic interplay between intracellular organelles is crucial to carry out several physiological activities, from modulation and decoding of Ca^2+^ signals to lipid synthesis and metabolism [40,41]. Specifically, the regions of close juxtaposition between organelle membranes host most of the enzymes/proteins mediating these cell functions. Moreover, the exchange of metabolites and/or information between organelles is favoured by their physical proximity, thus making contact areas, i.e., MAM domains in the case of ER–mitochondria contacts, the ideal hubs whereby key cellular tasks take place. Therefore, it is not surprising that alterations of organelle contacts and/or functionality have been associated with different pathological conditions, such as diabetes, cancer and neurodegeneration [27,40,42,43,44,45]. In particular, increasing evidence suggests that disturbances in ER–mitochondria connectivity are early events in neurodegenerative disorders such as Parkinson’s disease, amyotrophic lateral sclerosis and AD [46]. On this latter disorder, the majority of studies have highlighted an upregulated MAM functionality in different experimental models [14,18,19,39,47,48], although the underlying mechanisms, as well as its impacts on the neurodegenerative process, are still debated. For instance, we previously demonstrated that PS2 (in particular its FAD mutants), but not PS1, directly strengthens ER–mitochondria coupling [14,18], by interacting with MFN2 and tuning its negative activity on organelle juxtaposition [19,25]. Here, on the one hand, we further defined the PS2 protein domain mediating its functional interaction with MFN2 and, on the other, we generated a tool to correct the alterations in ER–mitochondria tethering triggered by FAD-PS2 mutants.

First, we found that PS2-CTF retains the activity of PS2, sustaining the efficiency of ER–mitochondria Ca^2+^ shuttling, while dampening the overall amplitude of IP3-linked cytosolic (and thus, mitochondrial) Ca^2+^ rises, likely as consequence of a reduced ER Ca^2+^ content. This result might be surprising, because different FAD-PS2 mutants, among those we previously demonstrated to potentiate ER–mitochondria coupling, display point mutations in the PS2-NTF. However, it should be considered that PS2-NTF and PS2-CTF originate from an autocleavage of full-length PS2, likely during its incorporation into γ-secretase [36]. Therefore, it is possible that while PS2-CTF modulates per se ER–mitochondria coupling, FAD-linked mutations enhance this activity by altering the subcellular localization of PS2-NTF/PS2-CTF, regardless of their position along the PS2 sequence. In line with this hypothesis, we previously observed that in the presence of FAD-PS2 mutants, both PS2-NTF and PS2-CTF are more enriched in MAM, compared to WT [19]. The reason underlying this distinct subcellular distribution of FAD-PS2 is unknown, but changes in the interaction with specific molecular partners might be involved.

Importantly, the PS2-CTF is composed of a large cytosolic loop followed by the remaining three TMD of the protein. Our data revealed that ER–mitochondria coupling is affected by the expression of the PS2-LOOP alone when targeted to the OMM (through an OMM-anchoring sequence), but not when expressed free in the cytosol. This further suggests that the enrichment of PS2 at specific subcellular domains is crucial to stoichiometrically favour its interaction with key molecular partners. Notably, we found that the OMM-targeted PS2-LOOP domain coimmunoprecipitates with MFN2, suggesting that it contains the minimal sequence mediating PS2-MFN2 interaction. Surprisingly, however, Mit-PS2-LOOP exerts opposite effects on ER–mitochondria tethering, compared to the entire PS2. This implies that, after binding to MFN2, PS2 performs an additional activity on this protein (such as the re-localization or prevention of MFN2 interaction with additional molecular partners), which is not conserved by Mit-PS2-LOOP. We speculated that Mit-PS2-LOOP might compete with PS2, in particular FAD-PS2, for MFN2 binding, thus hinting at the possibility to take advantage of this property to counteract the increased ER–mitochondria coupling observed in different FAD-PS2-expressing models. We tested this possibility by comparing the effects of Mit-PS2-LOOP expression in primary human fibroblasts from either a healthy donor or a FAD-PS2-N141I patient, wherein we previously reported, and we here confirmed (Figure 4), a larger physical and functional coupling between ER and mitochondria [19,32]. We indeed found that Mit-PS2-LOOP was able to rescue organelle tethering in FAD-PS2-N141I fibroblasts to levels similar to those observed in cells from a healthy donor (Figure 4), suggesting it might be particularly effective in the presence of FAD-PS2 mutants.

Importantly, to what extent the altered ER–mitochondria coupling observed in different AD models is involved in the neurodegenerative process remains an outstanding question. Nevertheless, increasing evidence suggests that pronounced alterations of lipid metabolism, such as hypercholesterolemia, are associated with AD onset and progression [49,50]. As a proof of this concept, the ε4 variant of apolipoprotein E (APOE), which works as a cholesterol/lipid transporter in the central nervous system, is considered the most frequent risk factor for AD development (reviewed in [51]). Remarkably, MAM are key regions for lipid synthesis [26], in particular for cholesterol metabolism. Indeed, free cholesterol is converted into cholesteryl esters (CEs) by acyl-CoA: cholesterol acyl transferase (ACAT1), an enzyme highly enriched in MAM [52], and key steps of phospholipid synthesis are typical activities localized to MAM [26]. In particular, an upregulated ACAT1-mediated esterification of excessive free cholesterol to CEs, leading to the accumulation of LDs in the cytosol, has been reported in different AD models [39,53] and associated with an increased MAM functionality [39]. Consistently, we confirmed an increased number and size of LDs in FAD-PS2-N141I fibroblasts and, remarkably, we found that correction of ER–mitochondria tethering by Mit-PS2-LOOP expression correlates with a rescue of their size and a tendency to a reduction in their number, to levels similar to those observed in control cells. Additional investigations will be required to test whether these observations are maintained in cell types with a higher pathological relevance (such as neurons differentiated from FAD patient-derived induced pluripotent stem cells) and their possible role on disease progression.

As to mitochondrial Ca^2+^ homeostasis, which is modulated by ER–mitochondria coupling, alterations in this signalling pathway have been reported in different neurodegenerative diseases, including AD [54]. In the specific case of FAD-PS2 mutants, upon IP3-dependent cell stimulation, a complex balance between a lower ER Ca^2+^ content (which weakens the amplitude of Ca^2+^ transfer to mitochondria) but a strengthened ER–mitochondria tethering (which increases the efficiency of ER–mitochondria Ca^2+^ shuttling), results in a decreased mitochondrial Ca^2+^ signal [14,18,19,20]. We associated this lower signal with a reduced mitochondrial ATP synthesis, affecting the overall cell bioenergetics [20]. Therefore, on this specific cell pathway, Mit-PS2-LOOP is unlikely to be helpful in the presence of FAD-PS2-mutants, because it further decreases mitochondrial Ca^2+^ peaks by decoupling mitochondria from the ER. In this pathological context, we foresee that a combination of treatments recovering ER Ca^2+^ content (such as by enhancing SERCA activity) with Mit-PS2-LOOP expression (which rescues organelle tethering) might be necessary to fully restore the ER–mitochondria axis.

We believe the modulation of ER–mitochondria juxtaposition is worthy of being harnessed as a promising pharmacological target in AD, because it has the potential to tune key pathways recently associated with disease onset and progression, such as altered mitochondrial Ca^2+^ homeostasis and lipid metabolism.

## Data Availability

Data are contained within the article or supplementary material.

## Supplementary Materials

The following are available online at https://www.mdpi.com/article/10.3390/cells10081968/s1, Figure S1: Mit-PS2-LOOP decreases ER–mitochondria coupling in HeLa cells without altering mitochondrial morphology; Table S1: Detailed statistical analysis.
