# Supplementary material for: Loosening ER–Mitochondria Coupling by the Expression of the Presenilin 2 Loop Domain
Source: Cells. 2021 Aug 3;10(8):1968. doi: 10.3390/cells10081968 (PMC8394530; doi:10.3390/cells10081968)
Supplement: Supplementary file 1 [file cells-10-01968-s001.zip › Cells-1303994-supplementary.pdf]

Supplementary Figure S1

HeLa cells

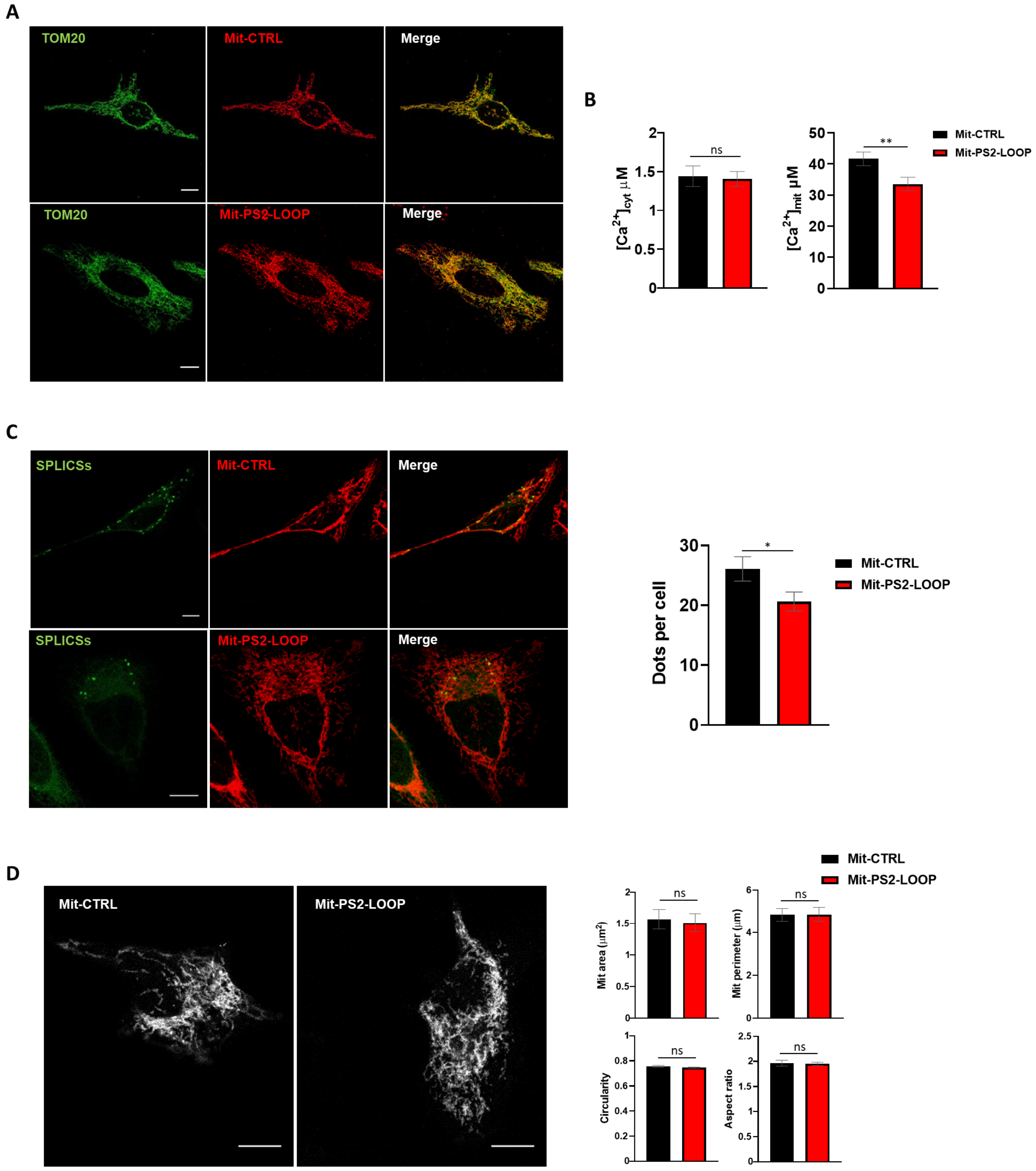

Supplementary Table S1

FIGURE 1		Test	p value			Test	p value			Test	p value
**Figure 1B**	CTRL vs CTRL pre-depleted	MW	0.0003	**Figure 1C**	CTRL vs CTRL pre-depleted	MW	0.0005				
	CTRL vs PS2-CTF	MW	0.0008		CTRL vs PS2-CTF	MW	0.001				
	CTRL vs PS2-WT	MW	0,0025		CTRL vs PS2-WT	MW	0.001				
	CTRL dep vs PS2-CTF	MW	0.0038		CTRL dep vs PS2-CTF	MW	0.0029				
	CTRL dep vs PS2-WT	MW	0.0003		CTRL dep vs PS2-WT	MW	<0.0001				
	PS2-CTF vs PS2-WT	MW	0.0864		PS2-CTF vs PS2-WT	MW	0.2123				
**FIGURE 2**											
**Figure 2E**	BK + CPA	MW	0.5996	**Figure 2F**	BK + CPA	TT	0.6826				
	FCS + CPA	TT	0.8327		FCS + CPA	MW	>0.9999				
**FIGURE 3**											
**Figure 3D**	BK+CPA	TT	0.5649	**Figure 3E**	BK+CPA	TT	0.0026	**Figure 3F**	CCE cyt	MW	0.9276
	FCS+CPA	TT	0.9791		FCS+CPA	TT	0.0175		CCE mit	MW	0.8801
**FIGURE 4**											
**Figure 4B**	CTRL: Mit-CTRL vs Mit-PS2- LOOP	MW	0.9406	**Figure 4C**	CTRL: Mit-CTRL vs Mit-PS2-LOOP	MW	0.4212	**Figure 4D**	CTRL: Mit-CTRL vs Mit-PS2-LOOP	MW	0.5499
	FAD-PS2: Mit-CTRL vs Mit-PS2-LOOP	MW	0.0359		FAD-PS2: Mit-CTRL vs Mit-PS2-LOOP	MW	0.9755		FAD-PS2: Mit-CTRL vs Mit-PS2-LOOP	MW	0.0283
	CTRL Mit-CTRL vs FAD-PS2 Mit-CTRL	MW	0.0456		CTRL: Mit-CTRL vs FAD-PS2: Mit-CTRL	MW	0.0332		CTRL: Mit-CTRL vs FAD-PS2: Mit-CTRL	MW	<0.0001
	CTRL Mit-PS2-LOOP vs FAD-PS2 Mit-PS2-LOOP	MW	0.0319		CTRL Mit-PS2-LOOP vs FAD-PS2: Mit-PS2-LOOP	MW	0.0206		CTRL: Mit-PS2-LOOP vs FAD-PS2: Mit-PS2-LOOP	MW	<0.0001
**Figure 4F**	CTRL: Mit-CTRL vs Mit-PS2-LOOP	MW	0.2704	**Figure 4G**	CTRL: Mit-CTRL vs Mit-PS2-LOOP	MW	0.1391				
	FAD-PS2: Mit-CTRL vs Mit-PS2-LOOP	MW	0.2293		FAD-PS2: Mit-CTRL vs Mit-PS2-LOOP	MW	<0.0001				
	CTRL: Mit-CTRL vs FAD-PS2: Mit-CTRL	MW	0.0214		CTRL: Mit-CTRL vs FAD-PS2: Mit-CTRL	MW	<0.0001				
	CTRL: Mit-PS2-LOOP vs FAD-PS2: Mit-PS2-LOOP	MW	0.0468		CTRL: Mit-PS2-LOOP vs FAD-PS2: Mit-PS2-LOOP	MW	0.0005				
					CTRL: Mit-CTRL vs FAD-PS2: Mit-PS2-LOOP	MW	<0.0001				
					CTRL: Mit-PS2-LOOP vs FAD-PS2: Mit-CTRL	MW	<0.0001				
**FIGURE S1**											
**Figure S1A**	CytAEQ	MW	0.763	**Figure S1B**	CTRL vs PS2	MW	0.0306	**Figure S1C**	MitArea	TT	0.797
	MitAEQ	TT	0.0096						Mit perimeter	TT	0.9583
									Circularity	TT	0.1968
									Aspect Ratio (AR)	MW	0.517
